# Supplementary material for: Transferring chemical and energetic knowledge between molecular systems with machine learning
Source: Commun Chem. 2023 Jan 13;6:13. doi: 10.1038/s42004-022-00790-5 (PMC9839695; doi:10.1038/s42004-022-00790-5)
Supplement: Supplementary file 2 — Supplementary Information [file 42004_2022_790_MOESM2_ESM.pdf]

Supplementary information

Supplementary Note 1 Metadynamics simulations

Metadynamics [28] is an enhanced sampling technique that employs an external bias potential applied to one or more degrees of freedom (also known as collective variables - CVs), and constructed as a sum of Gaussian functions. Each single Gaussian is defined by the following expression:

$$V(s,t) = \sum \omega e^{-\sum_{i=1}^{d} \frac{(s_i - s_i^0)^2}{2\sigma_i^2}} \tag{S.1}$$

where V is the total deposed bias, ω is the height of the Gaussian, d is the number of CVs where the potential is deposed, s is a given value in the CV, and σ is the width of the Gaussian. The bias potential acts as an enhancer, moving the system out of any minimum encountered during the simulation: the deeper the minimum, the greater amount of potential will be placed on a given position s along the CV, eventually overcoming all the barriers and entering in a semi-diffusive condition. Once the simulation has reached this level, and all possible states have been sampled, convergence is reached and the potential of mean force (PMF) can be reconstructed through the formula:

$$V(s) = -F(s) + C \tag{S.2}$$

where F represents the free-energy along the chosen CVs and C is a constant value. A variant of the original metadynamics is the "well-tempered" (WT) [3] approach:

$$V(s,t) = \sum \omega e^{\frac{-V(s,t)}{\Delta T}} e^{-\sum_{i=1}^{d} \frac{(s_i - s_i^0)^2}{2\sigma_i^2}} \tag{S.3}$$

where an exponential term re-weights the height of the Gaussian based upon how much potential has already been placed on the same point s at time t, and a parameter ΔT which regulates how fast the Gaussian height decreases. In particular, ΔT can also be seen as the difference in temperature between the hypothetical temperature felt by the enhanced CVs and the actual temperature of the simulation. To regulate this difference, the "biasfactor" is defined as $\gamma = (T + \Delta T)/T$, where T is the temperature of the system. In such a way, convergence is reached faster and errors in free-energy estimates are dumped out. The WT approach might require an experienced user and the final free-energy estimate can be calculated through a slightly revised formula:

$$V(s) = -\frac{\Delta T}{T + \Delta T} F(s) + C \tag{S.4}$$

The input data for all three systems discussed in the present work have been obtained from well-tempered metadynamics simulations. All production runs were carried out in vacuum conditions and with the Amber FF14SB force field [35]. We used the Sander program of Amber18 together with PLUMED2 in order to activate the metadynamics algorithm [11, 61]. In the following, we list the settings employed for the three investigated different systems:

- **alanine dipeptide**. This system has been thoroughly studied and it is known that the best CVs are the ϕ and ψ dihedral angles of alanine. During the metadynamics simulations, σ was set to 0.2 for ϕ and 0.3 for ψ, height was 1 kJ/mol, pace of 1000 steps, and biasfactor of 10;

- **tri-alanine**. We first sampled all possible structures using a general CV (i.e., RMSD of CA atoms), with a σ of 0.007, a height of 1.5 kJ/mol, a pace of 500, and a biasfactor of 20. Then, we performed a Time-lagged Independent Component Analysis (TICA) [36, 39] using the three couples of ϕ and ψ dihedrals in tri-alanine to construct two optimized CVs having the following construction:

$$CV1 = -0.0718\ \phi1 - 0.0550\ \psi1 - 0.9913\ \phi2 - 0.0100\ \psi2 + 0.0141\ \phi3\ - 0.0939\ \psi3 \tag{S.5}$$

$$CV2 = -0.0524\ \phi1 - 0.0931\ \psi1 + 0.0894\ \phi2 - 0.0973\ \psi2 + 0.9719\ \phi3 - 0.1630\ \psi3 \qquad \text{(S.6)}$$

These two CVs are the two eigenvectors with the highest spectral gap among the six constructed by TICA, and they allowed us to discriminate 9 different conformational states, discussed in the main paper. The metadynamics parameter employed for the optimized run were 0.05 of σ for both coordinates, 2 kJ/mol of height, pace of 500 steps, and biasfactor of 20;

- **deca-alanine**. We selected as CV the RMSD of the CA atoms of the residues, with a σ of 0.007, a height of 0.1 kJ/mol, and a pace of 500 steps.

It is worth noting that different CVs settings have been used in the diverse systems investigated. As alanine dipeptide is a single aminoacid, the two backbone dihedral angles have been used as CVs, while for the tri-alanine peptide a linear combination of all of its dihedrals was employed. Instead, deca-alanine represents a much more complex system due to its higher structural complexity. Endowed with 20 dihedral angles, it is difficult to obtain from TICA a low number of CVs composed by a weighted linear combination of descriptors of the system. Such a difficulty might compromise the convergence of the free-energy calculation. For this reason, for deca-alanine we decided not to reach free-energy convergence, but to use a more general RMSD-based CV and the phase space sampling power of metadynamics to ensure the visit of the energetically most relevant states without computing the associated free-energy (Fig. S.1).

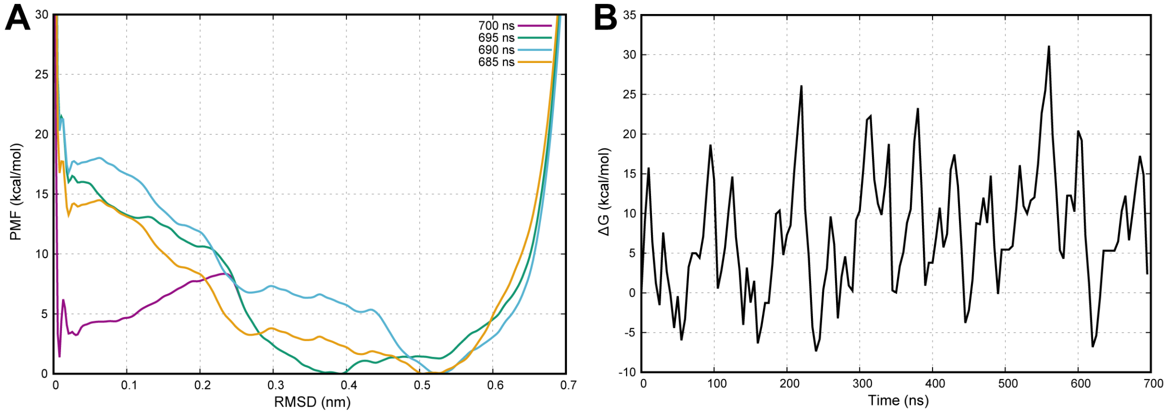

Figure S.1: Free energy surfaces of deca-alanine. A. Super-imposed free-energy surfaces at different times for the Metadynamics simulation of deca-alanine. B. Plot of the free-energy difference with respect to time between the conformations close to 0 nm and those at around 0.5 nm of RMSD.

Supplementary Note 2 Molecular dynamics simulations

Molecular Dynamics (MD) simulations were run for deca-alanine to sample structures around the ten different clusters that were obtained from metadynamics. The simulations were carried out in vacuum using the Amber FF14SB force field and the Sander algorithm of the Amber18 package [11, 35]. During the simulations, we set a constraint on the RMSD of the deca-alanine CA atoms at 0.1 nm to obtain 1000 additional structures distributed around the representative one. Each run lasted for 1 ns with a time-step of 2 fs, for a total of 10000 structures that were used for the classification task.

Supplementary Note 3 Cluster creation in MD simulations

For the deca-alanine system, a sample of structures were selected for the classification problem. These conformations were chosen by picking the ten most populated clusters over all the structures explored by the system during metadynamics. For this task, we employed the built-in functionality of Amber "cluster", which possesses several algorithms to associate a frame to a given family of structures [11]. In particular, we opted for the "hieragglo" bottom-up default algorithm, with an ϵ of 2, applied only on the C_α atoms of the peptide. A total of 475 clusters were obtained, and the ten most populated were selected to be used for the secondary structure prediction test described in the main text.

Supplementary Note 4 Classification of low and high free-energy conformations

To prepare training data for the model, we labelled any conformation with free-energy lower than 8 kJ/mol as low energy conformation, and everything else as high energy conformation. The classification performed by the model operate as follows:

- The model processes hypergraph representations of molecules and produces internal representations for the hypergraphs
- The internal representations are passed through a pooling layer, which assigns a fixed size vector representation to each hypergraph
- Each vector is inputted to a feed-forward neural network, assigning a probability $p \in [0, 1]$ of membership to the low-energy class
- Once all data are processed, we perform ROC analysis and compute the AUC, which gives us a robust measure of classification performance

Supplementary Note 5 p-values among clusters

Details of the p-values for the comparisons shown in Table S.1.

Table S.1: p-values for the test assessing whether two clusters are in significant disagreement in terms of free-energy predictions. Cluster IDs are shown on row and column headings. Yellow cells indicate that the respective predictions' agreement was not inline with our original estimate based on the family of clusters.

	0	1	2	3	4	5	6	7	8	9
0	N/A	1.96e-88	5.50e-55	3.63e-14	4.32e-77	1.02e-05	1.69e-09	1.89e-32	3.34e-18	2.51e-77
1	1.96e-88	N/A	7.80e-12	9.54e-54	0.0197	1.11e-66	1.67e-57	2.97e-37	3.71e-47	0.1582
2	5.50e-55	7.80e-12	N/A	2.18e-21	1.31e-07	2.04e-34	2.18e-28	2.65e-08	5.69e-16	1.55e-09
3	3.63e-14	9.54e-54	2.18e-21	N/A	8.60e-47	3.40e-05	0.0996	5.34e-07	0.1300	5.51e-46
4	4.32e-77	0.0197	1.31e-07	8.60e-47	N/A	1.86e-60	2.67e-49	7.06e-26	2.97e-37	0.4715
5	1.02e-05	1.11e-66	2.04e-34	3.40e-05	1.86e-60	N/A	0.0210	1.98e-17	1.97e-08	2.97e-64
6	1.69e-09	1.67e-57	2.18e-28	0.0996	2.67e-49	0.0210	N/A	7.36e-11	0.0012	5.91e-50
7	1.89e-32	2.97e-37	2.65e-08	5.34e-07	7.06e-26	1.98e-17	7.36e-11	N/A	0.0019	9.76e-27
8	3.34e-18	3.71e-47	5.69e-16	0.1300	2.97e-37	1.97e-08	0.0012	0.0019	N/A	3.04e-37
9	2.51e-77	0.1582	1.55e-09	5.51e-46	0.4715	2.97e-64	5.91e-50	9.76e-27	3.04e-37	N/A

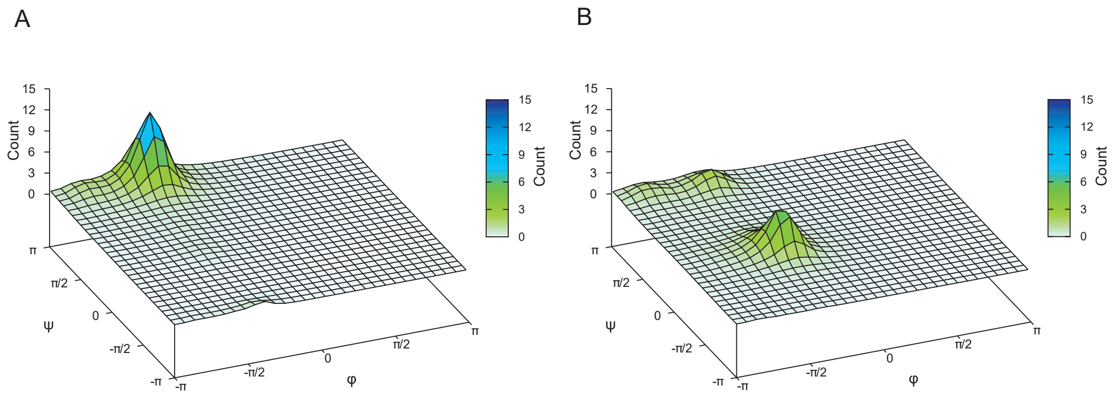

Figure S.2: Ramachandran plot for two different clusters of deca-alanine. Ramachandran plot for a distribution of 1000 structures of the last three terminal residues in cluster 2 and 4 (A and B, respectively). The different level of denaturation leads to a change in the response of the HNN model.
